# A novel dual-labeled small peptide as a multimodal imaging agent for targeting wild-type EGFR in tumors

**DOI:** 10.1371/journal.pone.0263474

**Published:** 2022-02-04

**Authors:** Myoung Hyoun Kim, Seul-Gi Kim, Dae-Weung Kim

**Affiliations:** 1 Department of Nuclear Medicine and Institute of Wonkwang Medical Science, Wonkwang University School of Medicine, Iksan, Jeollabuk-do, Korea; 2 Research Unit of Molecular Imaging Agent (RUMIA), Wonkwang University School of Medicine, Iksan, Jeollabuk-do, Korea; University of Magdeburg, GERMANY

## Abstract

The epidermal growth factor receptor (EGFR) is over-expressed in various human cancer. The over-expression of EGFR in tumors is an excellent target for the development of cancer imaging agents. In the present study, we developed Tc-99m SYPIPDT-GHEG-ECG-K-tetramethylrhodamine (SYPIPDT-ECG-TAMRA) as a molecular imaging agent targeting wild-type EFGR (wtEGFR)-positive tumor cells, and verified its feasibility as molecular imaging agent. SYPIPDT-ECG-TAMRA was synthesized using Fmoc solid-phase peptide synthesis. The radiolabeling of SYPIPDT-ECG-TAMRA with Tc-99m was accomplished using ligand exchange via tartrate. Cellular uptake and binding affinity studies were performed. *In vivo* gamma camera imaging, *ex vivo* imaging and biodistribution studies were performed using NCI-H460 and SW620 tumor-bearing murine models. After radiolabeling procedures with Tc-99m, Tc-99m SYPIPDT-ECG-TAMRA complexes were prepared at high yield (> 95%). The binding affinity value (K_d_) of Tc-99m SYPIPDT-ECG-TAMRA for NCI-H460 cells was estimated to be 76.5 ± 15.8 nM. In gamma camera imaging, the tumor to normal muscle uptake ratios of Tc-99m SYPIPDT-ECG-TAMRA increased with time (2.7 ± 0.6, 4.0 ± 0.9, and 6.2 ± 1.0 at 1, 2, and 3 h, respectively). The percentage injected dose per gram of wet tissue for the NCI-H460 tumor was 1.91 ± 0.11 and 1.70 ± 0.22 at 1 and 3 h, respectively. We developed Tc-99m SYPIPDT-ECG-TAMRA, which is dual-labeled with both radioisotope and fluorescence. *In vivo* and *in vitro* studies demonstrated specific uptake of Tc-99m SYPIPDT-ECG-TAMRA into wtEGFR-positive NCI-H460 cells and tumors. Thus, the results of the present study suggest that Tc-99m SYPIPDT-ECG-TAMRA is a potential dual-modality imaging agent targeting wtEGFR.

## Introduction

The human epidermal growth factor receptor (EGFR) is 1 of 4 members of the EGFR family of receptor tyrosine kinases [1]. The EGFR is a 1186 amino acid transmembrane glycoprotein, consisting of an extracellular domain, a transmembrane domain, and an intracellular tyrosine kinase domain [2]. EGFR is involved in a variety of cellular processes including cell growth, proliferation, differentiation, and apoptosis [2]. Furthermore, EGFR is over-expressed in various human cancers including lung, breast, head, neck, colon and rectum [3,4]. The EGFR signaling pathway is associated with angiogenesis, progression, metastasis and apoptosis of the tumor [2]. Thus, it is associated with survival, motility and resistance to treatments of patients [5].

Therefore, the over-expression of EGFR is an excellent target for developments of tumor imaging agent. EGFR antibody, Affibody molecule and EGFR tyrosine kinase inhibitor have been evaluated for candidate probes of EGFR imaging [6–8]. Anti-EGFR monoclonal antibodies conjugated MR contrast, F-18 labeled Affibody protein, C-11 erlotinib, Lu-177 nimotuzumab, or C-11 labeled 4-N-(3-bromoanilino)-6, 7-dimethoxyquinazoline, have been developed to target the EGFR of cancer [6–9]. Monoclonal antibodies that are able to bind specifically to EGFR were widely used for the development of imaging agents. However, a major drawback of these antibodies is their large size (~150 kDa). Large size of antibodies hinders efficient penetration into the targeted tumor and prevents rapid blood clearance. This results in high background activity and low target to non-target uptake ratio on image acquisition [10].

High binding affinity and specificity for targeted receptor is major characteristic of an ideal imaging probe. Also, an ideal imaging probe should be rapidly cleared from the blood in order to ensure adequate target-to-background ratio. In addition, high stability under physiological conditions, low immunogenicity and toxicity as well as easy production are all necessary for clinical translation [11]. Peptides are 2-dimensional, linear chains of amino acids and compared to antibodies, size of peptides is small (∼3–5 kDa). Therefore, peptides are easy to synthesize and modify, have higher cell membrane penetration and less immunogenicity [12]. Thus, EGFR-targeting peptides may be a better candidate for a new cancer imaging agent.

A small peptide, SYPIPDT was identified and tested for affinity and functional effect on wild-type EGFR (wtEGFR) by M Hamzeh-Mivehroud *et al*. [13]. The identified peptide was able to inhibit the epidermal growth factor-induced phosphorylation of EGFR in a concentration-dependent manner. In the present study, we developed Tc-99m SYPIPDT-GHEG-ECG-K-tetramethylrhodamine (SYPIPDT-ECG-TAMRA) as a molecular imaging agent targeting wtEGFR-positive tumor cells. Then, we evaluated the tumor imaging characteristics of the newly developed agent to provide proof of principle for the use of the dual-labeled imaging approach in mice with non–small cell lung cancer (NSCLC) xenografts. This newly developed agent targeting wtEGFR could provide molecular imaging of wtEGFR-positive tumor and fundamental information for quantitative analysis of certain EGFR mutations in future studies.

## Materials and methods

### Materials

Acetone, 1N-HCl, SnCl_2_, and sodium tartrate were purchased from Sigma Aldrich Korea (Seoul, Korea). Tc-99m pertechnetate was eluted from a commercial technetium generator (EnviroKorea, Daejeon, Korea) at our institution. A Radio-Cap®, capillary columns filled with silica materials (cat. FC-D1012, silica gel size = 38–75 μm; Futurechem, Seoul, Korea) were used for radio-chromatography. NCI-H460 (human non-small-cell lung cancer cell, wtEGFR-positive) and SW620 (human colon cancer cell, wtEGFR-negative) cell lines were obtained from the Korean Cell Line Bank (Seoul, Korea).

### Synthesis and characterization of SYPIPDT-ECG-TAMRA

SYPIPDT-ECG-TAMRA with purity greater than 90% was synthesized commercially by Peptron, Inc. (Daejeon, Korea). Briefly, peptides were synthesized using Fmoc solid-phase peptide synthesis (SPPS). The peptide-bound resin was treated with TAMRA-succinimidyl ester and diisopropylethylamine in N-methyl-2-pyrrolidone. The resulting compound was purified using reverse phase high-performance liquid chromatography (RP-HPLC) with a C18 analytical column (C18, 5 μm, 100Å column, 4.6 × 250 mm; Shimadzu, Kyoto, Japan). For elution, a linear gradient from 0 to 70% acetonitrile in water containing 0.1% trifluoroacetic acid (TFA) was used. Mass of synthesized peptide was analyzed by mass spectrometry (AXIMA-CFR, MALDI-TOF Mass Spectrometer, Shimadzu).

### Radiolabeling with Tc-99m

Radiolabeling of SYPIPDT-ECG-TAMRA with Tc-99m was done as described previously [14,15]. Briefly, a mixture of SYPIPDT-ECG-TAMRA (0.005 mg/ml in 300 μl nitrogen-purged water) and sodium tartrate (100 mg/ml in 300 μl nitrogen-purged water) was added in a microcentrifuge tube. This solution was mixed with Tc-99m pertechnetate (1.0 ml, about 1,110 MBq) and SnCl_2_ (1 mg/ml in 30 μl nitrogen-purged 0.01 M HCl). The solution was heated at 95°C for 15 min and cooled at room temperature. Characterization of radiolabeled Tc-99m SYPIPDT-ECG-TAMRA was done by a HPLC consisting of Gilson 321 HPLC pumps (Gilson, Inc., Middleton, WI, USA), a Bioscan FC-1000 radiodetector (Bioscan, Inc., Washington D.C., USA), Trilution LC software (Gilson, Inc.), and an YMC-Triart C18 column (4.6 × 100 mm; YMC, Kyoto, Japan). Solvent A and B was 0.1% TFA in water and acetonitrile, respectively. At a flow rate of 1 ml/min, a linear gradient from 0 to 35% solvent B over 35 min was employed. An ultraviolet detector (230 nm) and gamma radiodetector were used for the monitoring.

To evaluate the radiochemical stability of the Tc-99m peptide complex, the labeled complex (0.1 ml) was incubated with 1 ml of saline at room temperature and freshly collected human serum at 37°C. Samples were collected at 30 min and at 1, 3, and 24 h and were analyzed using capillary column with saline (R_f_ of Tc-99m SYPIPDT-ECG-TAMRA and free pertechnetate = 0.9–1.0; R_f_ of colloid = 0.0–0.1) and acetone (R_f_ of free pertechnetate = 0.9–1.0; R_f_ of Tc-99m SYPIPDT-ECG-TAMRA and colloid = 0.0–0.1) as mobile phases. All experiments were conducted in triplicate (n = 3).

### *In vitro* receptor binding affinity

NCI-H460 and SW620 cells were cultured in a humidified atmosphere with 5% carbon dioxide at 37°C. The cells were maintained in Dulbecco modified Eagle medium supplemented with 10% fetal bovine serum, streptomycin (100 μg/ml), and penicillin (100 U/ml). The cells were used for experiments and studies once about 80% confluence had been reached.

The binding affinities of Tc-99m SYPIPDT-ECG-TAMRA for NCI-H460 and SW620 cells were evaluated by saturation binding studies as described previously [16]. Briefly, NCI-H460 and SW620 cells were plated into 96-well plates at a density of 1 × 10^4^ cells/well and cultured overnight. Cells were washed twice (5 min each) using ice-cold binding buffer (25 mM HEPES and 1% bovine serum albumin). A concentration gradient (0–2,000 nM) of Tc-99m SYPIPDT-ECG-TAMRA was added to the wells and the cells were maintained at 37°C for 1 h. The total volume of each well was 200 μl. After cells were washed twice using ice-cold binding buffer, the cells of each well were obtained for gamma counting. Radioactivities of collected cells were measured using a 1480 Wizard 3 gamma counter (PerkinElmer Life and Analytical Sciences, Wallingford, CT, USA). The binding affinity value (K_d_) was calculated by non-linear regression models of GraphPad Prism software version 5.03 (GraphPad Software, La Jolla, CA, USA). Each data point is the average value of four determinations.

### Cellular uptake using confocal microscopy

NCI-H460 and SW620 cells (1 × 10^5^ cells) were cultured on the top of a cover-slip slide for 24 h at 37°C. The medium was replaced with fresh serum free medium (500 μL) containing Tc-99m SYPIPDT-ECG-TAMRA (200 nM). Cells were then incubated at 37°C for 1 h. These cells were rinsed three times with phosphate-buffered saline (PBS). The cell were stained with anti-human wtEGFR antibody (cat. 4267S; Cell Signaling Technology, Danvers, MA, USA) and Alexa Fluor® 488-conjugated goat anti-rabbit secondary antibody (cat. 111-545-003, 1:100 dilution; Jackson Immuno Research Inc., West Grove, PA, USA). The slides were covered with fluorescent mounting medium (Dako, Glostrup, Denmark) and closed with cover glasses. The confocal microscopic study was performed with a FV1200 confocal microscope (Olympus, Pittsburgh, PA, USA) with a 100× oil immersion lens.

### Murine models with tumors

Six-week-old female homozygous athymic BALB/c nu/nu mice (weighing 16–18 g) were purchased from Damul Science (Daejeon, Korea) and kept in cages. They were provided free access to food and tap water. After one week of adaptation to laboratory conditions, the mice were subcutaneously inoculated with 1 × 10^7^ NCI-H460 (wtEGFR-positive) and SW620 (wtEGFR-negative, as an internal reference) cells (in 0.1 ml PBS) in both sides of the anterior chest region, respectively. The tumors were allowed to grow to about 300 to 400 mm^3^ in volume. Tumor volume was calculated using the formula: V (mm^3^) = 0.5 × *a* × *b*^2^, where *a* and *b* represent the long and perpendicular short diameters of the tumor by caliper measurements. Approximately 21 days after inoculation, gamma camera imaging and biodistribution studies were performed.

### *In vivo* gamma camera imaging

Intra-peritoneal injection of ketamine (60 mg/kg) and xylazine (5 mg/kg) was used for anesthesia of tumor-bearing mice. 55.5 MBq (200 nM in 150 μL) of Tc-99m SYPIPDT-ECG-TAMRA was intravenously administered via the tail vein. At 1, 2, and 3 h after injection, *in vivo* imaging was performed using a gamma camera (Vertex; ADAC Laboratories, Milpitas, CA, USA) equipped with a 3 mm pinhole collimator, a window setting of 140 keV, and a width of 20%. Acquisition times were 120 s and images were digitally stored in a 512 × 512 matrix size. Regions of interest (ROIs, 15 × 15 pixel sized) were drawn at the tumors on chest walls. Additional ROIs were drawn at the left arm muscle for evaluating normal muscle uptake (n = 5). The mean counts per pixel within the ROIs were obtained and target to non-target ratios were calculated.

### *Ex vivo* fluorescent imaging and immunohistochemical staining

Following the *in vivo* gamma camera imaging study, mice were sacrificed by cervical dislocation after anesthesia by intraperitoneal injection of 100 mg/kg bodyweight ketamine (n = 5). Tumor tissue and several different organs were excised and an *ex vivo* fluorescent imaging study was performed using a fluorescence imaging system (VISQUE™ InVivo Smart-LF, Vieworks, Anyang, Korea). The emission band of 520 to 675 nm was used for TAMRA (peak absorbance and peak emission wavelength = 565 and 580 nm, respectively). Exposure time was 2.0 s per image. The images were analyzed using the dedicated software.

After the *ex vivo* imaging study, tumor tissues were snap-frozen in liquid nitrogen. Frozen tumor tissues were cut into slices (10 μm in thickness). After drying at room temperature, slices were fixed with ice-cold acetone for 10 min and then air dried at room temperature for 20 min. Tumor sections were maintained with 5% goat serum for 30 min at room temperature for blocking of nonspecific binding. Then, tumor sections were stained with anti-human wtEGFR antibody (cat. 4267S; Cell Signaling Technology, Danvers, MA, USA) for 1 h at room temperature. After being rinsed with PBS buffer, tumor slides were incubated with Alexa Fluor® 488-conjugated goat anti-rabbit secondary antibody (cat. 111-545-003, 1:100 dilution; Jackson Immuno Research Inc., West Grove, PA, USA). After being washed three times with PBS, tumor slides covered using Prolong^®^ Gold Antifade Reagent with 4’,6-diamidino-2-phenylindole (DAPI; Invitrogen, Carlsbad, CA, USA) and closed with cover glasses. Confocal microscopic study was performed using a FV1200 confocal microscope (Olympus) with a 60× oil immersion lens.

### Biodistribution studies

Tissues of tumors and selected organs were weighed and collected into pre-weighed gamma counter tubes. The radioactivity of the tissues were counted using a gamma counter (1480 Wizard 3; PerkinElmer Life and Analytical Sciences) and counts per minute were decay-corrected. Results are expressed as a percentage injected dose per gram of wet tissue (%ID/g). Total activities injected per animal were calculated by evaluating the difference between pre-injection syringe counts and remaining syringe counts after injection.

### Statistical analysis

SPSS version 18.0 (IBM Corp., Armonk, NY, USA) was used for analyzing the data. Data are presented as means ± standard deviation (SD). Target to non-target ratios of gamma camera imaging and competition studies were compared using one-way analysis of variance (ANOVA) and appropriate post-hoc tests. For all tests, *p*-value < 0.05 indicated statistical significance.

### Ethical considerations

All animal experiments were performed in accordance with the guidelines of the Wonkwang University School of Medicine Committee to minimize the pain and sacrifice of the animals.

## Results

### Synthesis of SYPIPDT-ECG-TAMRA and radiolabeling with Tc-99m

**Fig 1:**
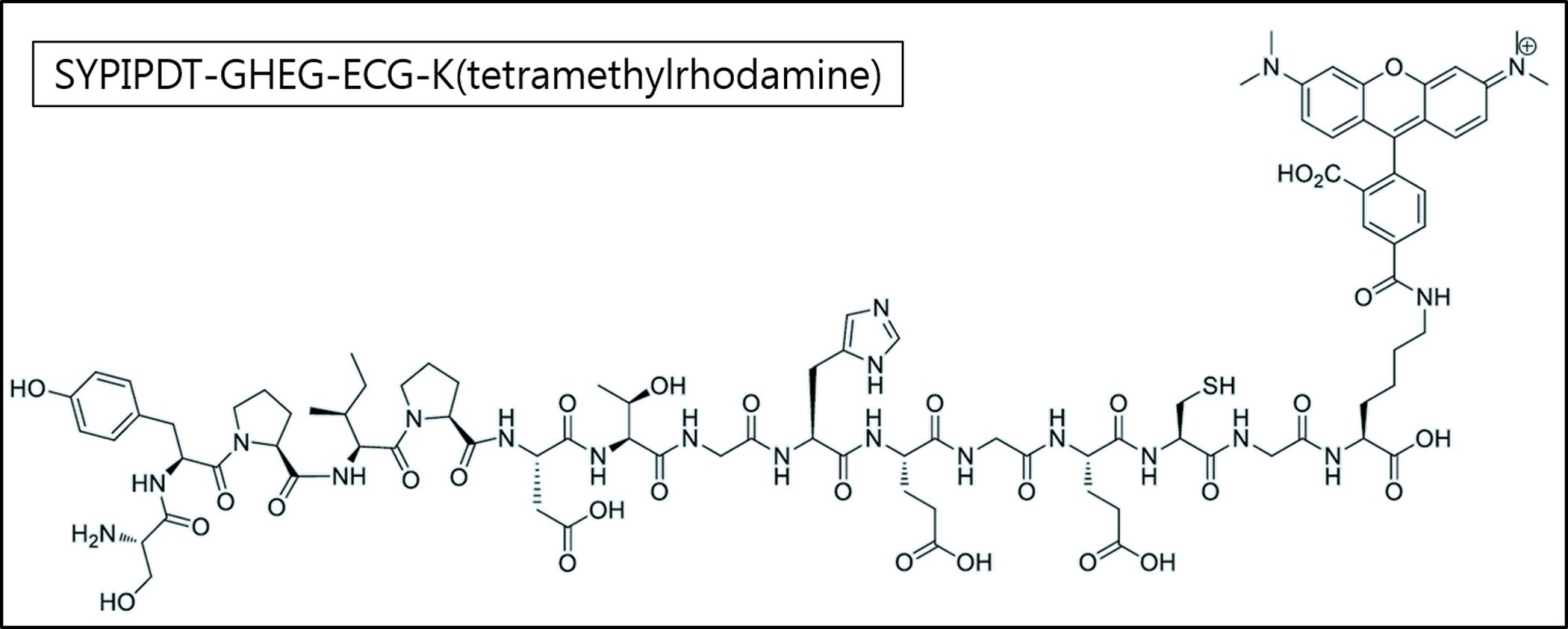
Chemical structure of SYPIPDT-GHEG-ECG-K-tetramethylrhodamine (SYPIPDT-ECG-TAMRA).

SYPIPDT-ECG-TAMRA (C_92_H_121_N_20_O_29_S_1_, Mol. Wt.: 2001.11) was successfully synthesized using Fmoc SPPS (Fig 1). After Tc-99m radiolabeling, a single radio-compound was detected by RP-HPLC (retention time = 7.1 min, S1 Fig). The Tc-99m SYPIPDT-ECG-TAMRA complex was prepared in high yield (> 95%) and showed high stability in saline and serum. The intact percentages of Tc-99m SYPIPDT-ECG-TAMRA incubated in saline as measured by capillary column filled with silica materials were 95.1 ± 0.6, 94.7 ± 1.3, 93.9 ± 1.1, and 92.1 ± 1.4% at 30 min, 1 h, 3 h, and 24 h, respectively. The intact percentages of Tc-99m SYPIPDT-ECG-TAMRA incubated in serum were 94.7 ± 0.8, 93.5 ± 0.7, 93.1 ± 1.0, and 91.5 ± 1.2% at 30 min, 1 h, 3 h, and 24 h, respectively.

### *In vitro* receptor binding affinity and cellular uptake

**Fig 2:**
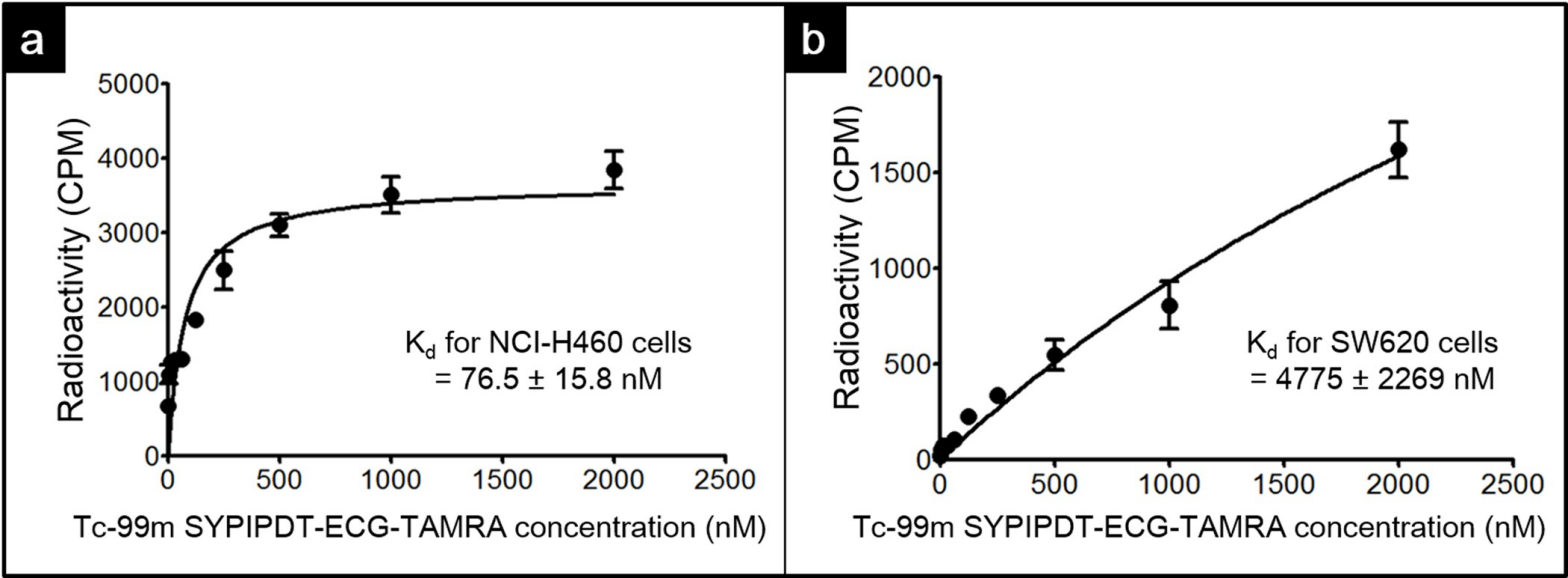
Cell binding affinity curves of Tc-99m SYPIPDT-ECG-TAMRA for NCI-H460 (a) and SW620 (b) cells.

The K_d_ of Tc-99m SYPIPDT-ECG-TAMRA for NCI-H460 cells was estimated to be 76.5 ± 15.8 nM (Fig 2A), which was a suitable affinity for wtEGFR-targeted imaging, and the B_max_ was determined 3652 ± 189 fmol/mg protein. In contrast, the binding of Tc-99m SYPIPDT-ECG-TAMRA for SW620 cells did not become saturated. The K_d_ value was estimated to be 4775 ± 2269 nM (Fig 2B), which was significantly higher than that for NCI-H460 cells (*p* < 0.005). These results demonstrate that Tc-99m SYPIPDT-ECG-TAMRA shows a high affinity for wtEGFR-specific uptake.

**Fig 3:**
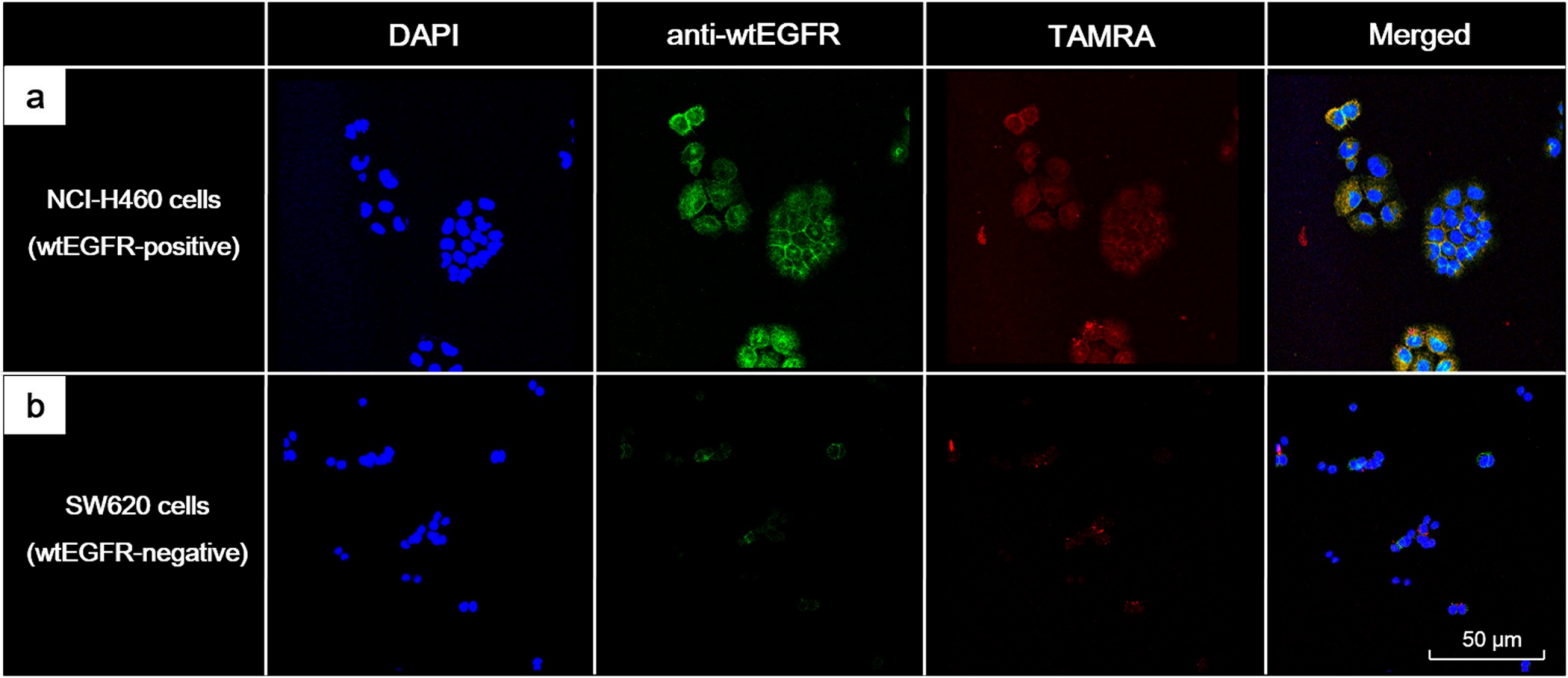
(a) Confocal microscopy images of wtEGFR-positive NCI-H460 cells incubated with Tc-99m SYPIPDT-ECG-TAMRA showed strong fluorescence activity (red) on the cell membrane. On the merged image of DAPI (blue), anti-wtEGFR (green) and SYPIPDT-ECG-TAMRA (red) channels, the TAMRA activity could be correlated with anti-wtEGFR activity. (b) In contrast, the fluorescence activities of SYPIPDT-ECG-TAMRA (red) and anti-wtEGFR (green) were barely detectable in wtEGFR-negative SW620 cells.

Confocal microscopy images of NCI-H460 cells incubated with SYPIPDT-ECG-TAMRA revealed the strong fluorescence activity of TAMRA (red) on the cell membrane. On the merged image of DAPI (blue), anti-wtEGFR (green) and SYPIPDT-ECG-TAMRA (red) channels, the activity of SYPIPDT-ECG-TAMRA could be correlated with anti-wtEGFR signal (Fig 3A). In contrast, the fluorescence activities of SYPIPDT-ECG-TAMRA and anti-wtEGFR were barely detectable in SW620 cells (Fig 3B).

### *In vivo* gamma camera imaging

**Fig 4:**
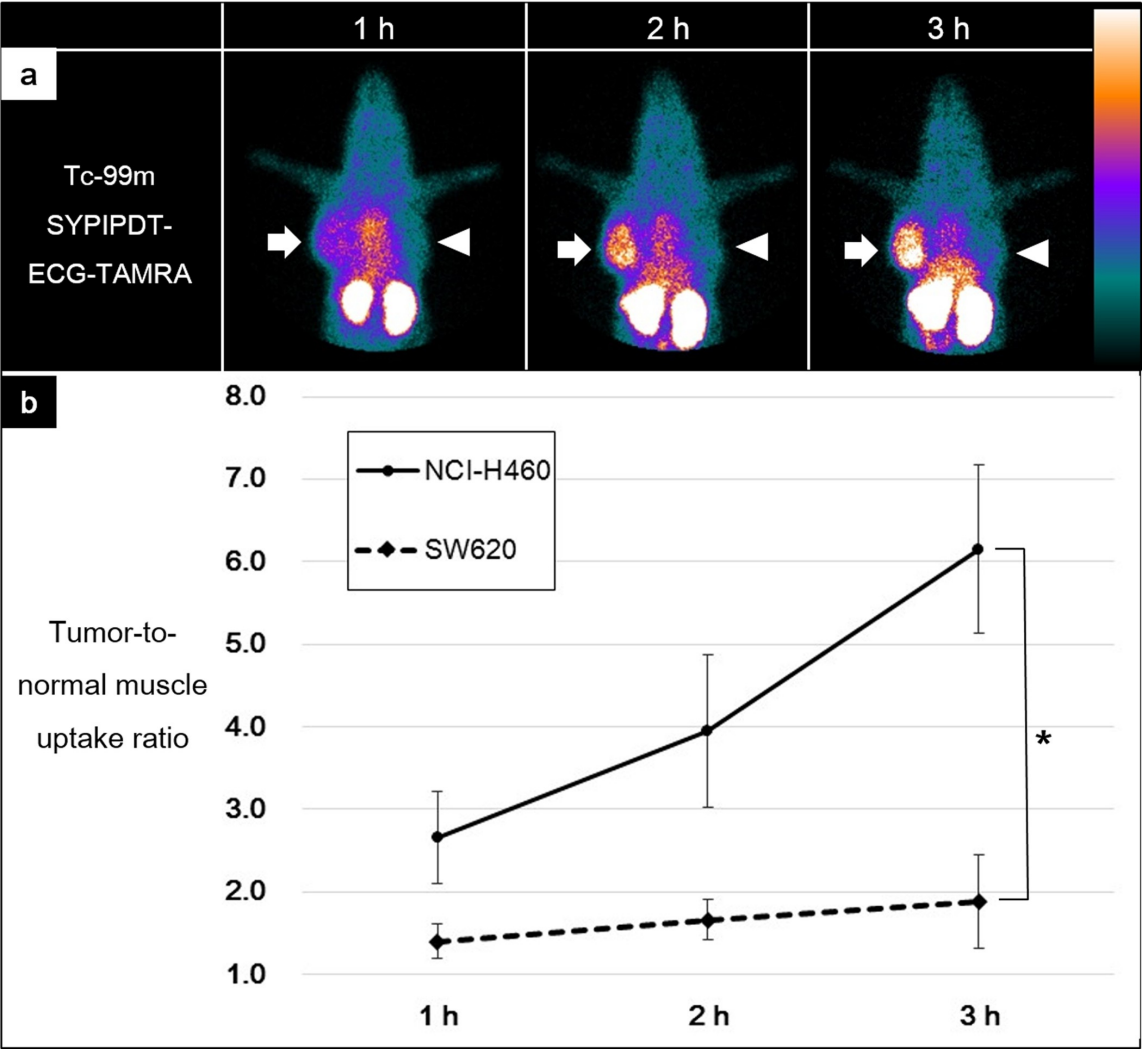
(a) After injection of Tc-99m SYPIPDT-ECG-TAMRA, serial gamma camera imaging of tumor-bearing mice revealed substantial uptake in the NCI-H460 tumors (arrows). In contrast, SW620 tumors (arrow heads) showed relatively low uptake of Tc-99m SYPIPDT-ECG-TAMRA. (b) At 1, 2 and 3 h after injection of Tc-99m SYPIPDT-ECG-TAMRA, tumor-to-normal muscle uptake ratios of NCI-H460 tumors were significantly higher than those of SW620 tumors (*p* < 0.05, asterisks).

In mice, the highest activity was observed in the kidneys, suggesting that Tc-99m SYPIPDT-ECG-TAMRA was mainly excreted through the renal system. Tc-99m SYPIPDT-ECG-TAMRA accumulated substantially in wtEGFR-positive NCI-H460 tumors (Fig 4A, arrows). The tumor to normal muscle uptake ratio of Tc-99m SYPIPDT-ECG-TAMRA increased with time (2.7 ± 0.6, 4.0 ± 0.9, and 6.2 ± 1.0 at 1, 2, and 3 h, respectively). In contrast, Tc-99m SYPIPDT-ECG-TAMRA did not significantly accumulate in wtEGFR-negative SW620 tumors (Fig 4A, arrow heads). The SW620 tumor to normal muscle uptake ratios (1.4 ± 0.2, 1.7 ± 0.2, and 1.9 ± 0.6 at 1, 2, and 3 h, respectively) were significantly lower than those of the NCI-H460 tumors (*p* < 0.05, asterisk, Fig 4B).

### *Ex vivo* fluorescent imaging and immunohistochemical staining

**Fig 5:**
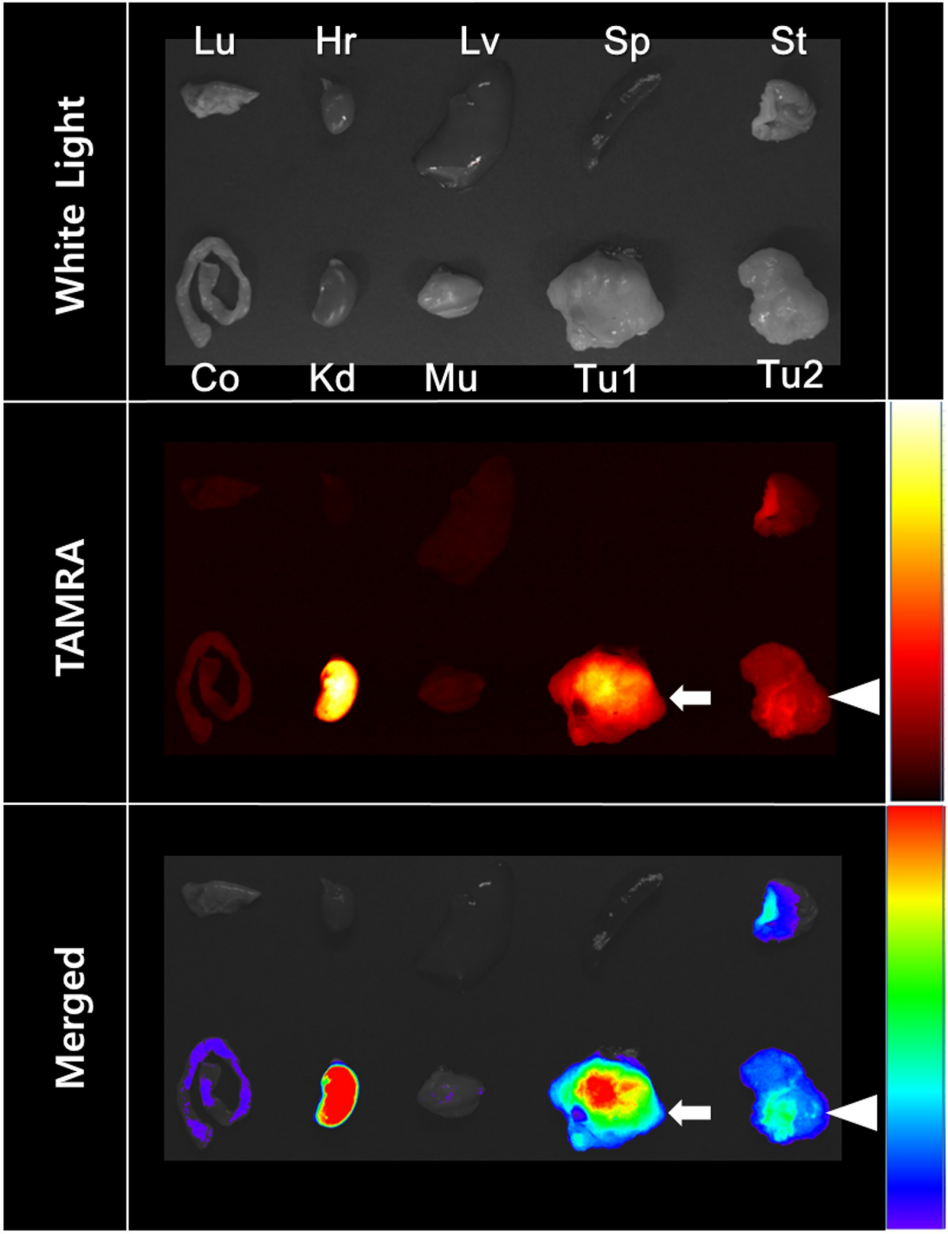
On *ex vivo* fluorescent images of excised organs, NCI-H460 tumors showed significantly higher fluorescent activity of Tc-99m SYPIPDT-ECG-TAMRA (arrows) than SW620 tumors (arrow heads). (Lu, lung; Hr, heart; Lv, liver; Sp, spleen; St, stomach; Co, colon; Kd, kidney; Mu, muscle; Tu1, NCI-H460 tumor; Tu2, SW620 tumor).

On the *ex vivo* fluorescent image, high fluorescent activity of TAMRA was detected in the kidney among the healthy organs (Fig 5). NCI-H460 tumor (arrows) showed significantly higher fluorescent activity of Tc-99m SYPIPDT-ECG-TAMRA than SW620 tumor (arrow heads). These observations were strongly correlated with the *in vivo* gamma camera images.

**Fig 6:**
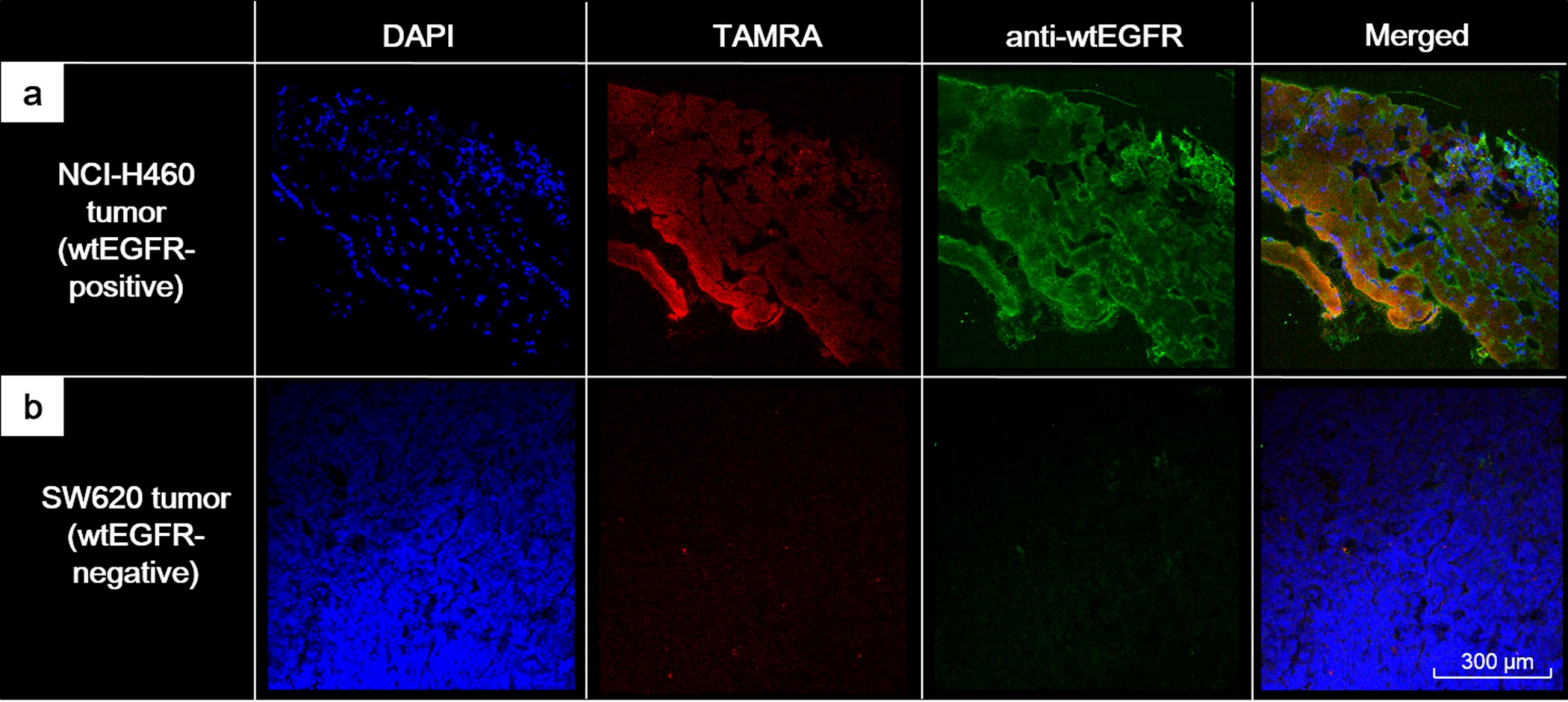
(a) On confocal microscopy with immunohistochemical staining, evident fluorescent TAMRA activities of Tc-99m SYPIPDT-ECG-TAMRA were detected within NCI-H460 tumor tissue, consistent with anti-wtEGFR antibody staining. (b) Fluorescent activity of TAMRA and anti-wtEGFR antibody was barely detected in the SW620 tumor tissue.

Confocal microscopy with immunohistochemical staining detected significant Tc-99m SYPIPDT-ECG-TAMRA fluorescence within NCI-H460 tumor tissue. The fluorescence was correlated with the fluorescence activity of anti-wtEGFR antibody (Fig 6A). However, the fluorescent activity of TAMRA and anti-wtEGFR antibody was barely detected in SW620 tumor tissue (Fig 6B).

### Biodistribution studies

The %ID/g biodistribution values at 1 and 3 h after injection of Tc-99m SYPIPDT-ECG-TAMRA are summarized in Table 1. At 1 h, the kidneys showed the highest activity and relatively high activities were observed in the blood, lung and liver. At 3 h, the activities of normal organs except the kidneys and liver decreased substantially, representing the fast washout of unbound Tc-99m SYPIPDT-ECG-TAMRA. Values of %ID/g for NCI-H460 tumors (1.91 ± 0.11 and 1.70 ± 0.22 at 1 and 3 h, respectively) were significantly higher than those of SW620 tumors (1.27 ± 0.43 and 0.54 ± 0.21 at 1 and 3 h, respectively). In NCI-H460 tumors, the tumor to normal muscle ratios of the %ID/g values were 2.45 ± 0.22 and 7.13 ± 2.00 for Tc-99m SYPIPDT-ECG-TAMRA at 1 and 3 h, respectively.

**Table 1:** Biodistribution data 1 and 3 h after injection of Tc-99m SYPIPDT-ECG-TAMRA in mice bearing NCI-H460 and SW620 tumors.

Organs	Mean %ID/g (SD)	
	1 h	3 h
Lungs	2.23 (0.63)	0.61 (0.10)
Heart	1.43 (0.38)	0.45 (0.17)
Blood	3.22 (1.17)	0.87 (0.28)
Liver	1.90 (0.53)	1.07 (0.20)
Stomach	1.68 (0.54)	0.37 (0.20)
Colon	1.11 (0.38)	0.42 (0.12)
Kidneys	21.05 (4.03)	12.40 (2.02)
Muscles	0.79 (0.12)	0.25 (0.08)
NCI-H460 tumor (wtEGFR-positive)	1.91 (0.11)	1.70 (0.22)
SW620 tumor (wtEGFR -negative)	1.27 (0.43)	0.54 (0.21)

## Discussion

Molecular imaging is an attractive method for assaying EGFR expression in tumors. It could provide visualization, non-invasive characterization, and quantitative measurement of biologic processes at the cellular or subcellular level [17]. Several imaging modalities including PET, SPECT, and MR have been introduced to evaluate EGFR expression [17]. Non-invasive molecular imaging agents, such as anti-EGFR monoclonal antibodies conjugated MR constrast, Tc-99m 8B6 Nanobody, F-18 Affibody protein, C-11 erlotinib, Lu-177 nimotuzumab, and C-11 4-N-(3-bromoanilino)-6, 7-dimethoxyquinazoline, have been developed for evaluating EGFR status of tumor [6–10].

A major drawback of these imaging agents is their large size, which hinders efficient penetration into the targeted tissue and prevents rapid clearance from the non-targeted tissues. Therefore, efforts have been made to reduce the size of the targeting agents. Monoclonal antibody fragments such as Fab′, scFv, Affibody and Nanobody, have been investigated [18]. However, the antibody fragments show reduced binding affinity for their target compared to the parental antibody, and their stability also decreases over time [19]. In the present study, we tried to overcome these drawbacks by utilizing a small peptide, SYPIPDT, as a targeting ligand. M Hamzeh-Mivehroud *et al*. had identified this peptide ligands for wtEGFR using a 7-mer peptide displaying phage display. The SYPIPDT peptide had diminished the increased EGF-dependent phosphorylation of EGFR by 44%. This inhibition was concentration dependent and the inhibitory effects were specific for EGFR [13]. Likewise, incorporation of ECG peptide and TAMRA could affect the binding affinity of SYPIPDT peptide. However, the rapid clearance of Tc-99m SYPIPDT-ECG-TAMRA from the non-targeted tissues could improve target-to-background ratio and shorten the injection-to-scan time.

Another major drawback of several imaging agents for targeting EGFR is high hepatic uptake. High hepatic radioactivity could reduce overall image quality, disturb assessments of tumors in this region and increase the radiation exposure. A high expression level of hepatic EGFR could be responsible for the significant accumulation of radiolabeled EGF in the liver [20,21]. Moreover, the physicochemical properties of these imaging agents, such as large size and lipophilicity could cause high hepatic uptake [10]. In the present study, Tc-99m SYPIPDT-ECG-TAMRA showed low hepatic uptake (1.07 ± 0.20%ID/g at 3 h after injection) compared to those of previously investigated EGFR-targeting imaging agents. The overall charge, small size and hydrophilicity of Tc-99m SYPIPDT-ECG-TAMRA might lead to relatively low hepatic uptake.

It has become necessary to develop a noninvasive imaging agent for detecting EGFR mutation status *in vivo*. EGFR mutation targeted imaging agents could enable selecting patients potentially responsive to EGFR-targeted treatment and monitoring changes in EGFR mutation status during treatment [22]. Thus, optimal clinical treatments could be offered for the patients. EGFR-targeted molecular imaging is an attractive tool for evaluating *in-vivo* EGFR mutation status because it can non-invasively acquire the molecular and genomic characteristics of the tumor and whole-body [22]. A molecular imaging agent targeting not EGFR mutation but wtEGFR is also important to assess the EGFR mutation status, because wild-type cells are mixed with mutant cells in varying proportions, referring intratumoral heterogeneity [23,24].

We attached GHEG-ECG-K-TAMRA to the SYPIPDT peptide sequence. We inserted a histidine-containing sequence, GHEG, between the SYPIPDT peptide and ECG-K-TAMRA as a linker or spacer. King R *et al*. reported that the histidine could displace one ligand from another [25]. Thus, additional distance between two ligands is secured and this could reduce the effect or disturbance of chelating ligand on the targeting ability of the SYPIPDT peptide. The ECG sequence, a tripeptide including multiple nitrogen atoms and one sulfur atom, showed strong and stable chelation with Tc-99m. It could be considered as a good candidate for a Tc-99m chelating ligand [14,15]. In addition, the fluorescent dye, TAMRA was incorporated for *ex vivo* optical imaging and *in vitro* immunohistochemical studies. To the best of our knowledge, this is the first study to develop a wtEGFR-targeting multimodality imaging agent containing both Tc-99m and fluorescent dye.

## Conclusions

We developed Tc-99m SYPIPDT-ECG-TAMRA which is dual-labeled with both radioisotope and fluorescence. *In vivo* and *in vitro* studies demonstrated specific uptake of Tc-99m SYPIPDT-ECG-TAMRA into wtEGFR-positive NCI-H460 cells and tumors. Also, tumor uptake of Tc-99m SYPIPDT-ECG-TAMRA was correlated with *ex vivo* fluorescent imaging and immunohistochemical staining studies. Taken together, the results of the present study suggest that Tc-99m SYPIPDT-ECG-TAMRA is a potential dual-modality imaging agent targeting wtEGFR.

## Supporting information

S1 Fig Radio high-performance liquid chromatography (HPLC) analysis of Tc-99m SYPIPDT-GHEG-ECG-K-tetramethylrhodamine (SYPIPDT-ECG-TAMRA) for 30 min after labeling. The retention time of labeled peptide was from 6.5 to 8.0 min and for mixture of Tc-99m pertechnetate and Tc-99m tartrate was 0.5–1 min.
(TIF)

## References

[1] Yarden Y, Sliwkowski MX. Untangling the ErbB signalling network. Nature reviews Molecular cell biology. 2001;2(2):127–37. Epub 2001/03/17. doi: 10.1038/35052073 . 11252954

[2] Yarden Y. The EGFR family and its ligands in human cancer. signalling mechanisms and therapeutic opportunities. Eur J Cancer. 2001;37 Suppl 4:S3-8. Epub 2001/10/13. doi: 10.1016/s0959-8049(01)00230-1 . 11597398

[3] Hirsch FR, Varella-Garcia M, Bunn PA Jr., Di Maria MV, Veve R, Bremmes RM, et al. Epidermal growth factor receptor in non-small-cell lung carcinomas: correlation between gene copy number and protein expression and impact on prognosis. Journal of clinical oncology: official journal of the American Society of Clinical Oncology. 2003;21(20):3798–807. Epub 2003/09/04. doi: 10.1200/JCO.2003.11.069 . 12953099

[4] Walker RA, Dearing SJ. Expression of epidermal growth factor receptor mRNA and protein in primary breast carcinomas. Breast cancer research and treatment. 1999;53(2):167–76. Epub 1999/05/18. doi: 10.1023/a:1006194700667 . 10326794

[5] Ciardiello F, Tortora G. EGFR antagonists in cancer treatment. The New England journal of medicine. 2008;358(11):1160–74. Epub 2008/03/14. doi: 10.1056/NEJMra0707704 . 18337605

[6] Vera DR, Eigner S, Henke KE, Lebeda O, Melichar F, Beran M. Preparation and preclinical evaluation of 177Lu-nimotuzumab targeting epidermal growth factor receptor overexpressing tumors. Nuclear medicine and biology. 2012;39(1):3–13. Epub 2011/10/01. doi: 10.1016/j.nucmedbio.2011.07.001 . 21958849

[7] Miao Z, Ren G, Liu H, Qi S, Wu S, Cheng Z. PET of EGFR expression with an 18F-labeled affibody molecule. Journal of nuclear medicine: official publication, Society of Nuclear Medicine. 2012;53(7):1110–8. Epub 2012/06/13. doi: 10.2967/jnumed.111.100842 ; PubMed Central PMCID: PMC4214858. 22689926 PMC4214858

[8] Memon AA, Jakobsen S, Dagnaes-Hansen F, Sorensen BS, Keiding S, Nexo E. Positron emission tomography (PET) imaging with [11C]-labeled erlotinib: a micro-PET study on mice with lung tumor xenografts. Cancer research. 2009;69(3):873–8. Epub 2009/01/22. doi: 10.1158/0008-5472.CAN-08-3118 . 19155297

[9] Shazeeb MS, Sotak CH, DeLeo M 3rd, Bogdanov A Jr. Targeted signal-amplifying enzymes enhance MRI of EGFR expression in an orthotopic model of human glioma. Cancer research. 2011;71(6):2230–9. Epub 2011/01/20. doi: 10.1158/0008-5472.CAN-10-1139 ; PubMed Central PMCID: PMC3059397. 21245103 PMC3059397

[10] Huang L, Gainkam LO, Caveliers V, Vanhove C, Keyaerts M, De Baetselier P, et al. SPECT imaging with 99mTc-labeled EGFR-specific nanobody for in vivo monitoring of EGFR expression. Molecular imaging and biology. 2008;10(3):167–75. Epub 2008/02/26. doi: 10.1007/s11307-008-0133-8 . 18297364

[11] Sun X, Li Y, Liu T, Li Z, Zhang X, Chen X. Peptide-based imaging agents for cancer detection. Advanced drug delivery reviews. 2017;110–111:38–51. Epub 2016/06/22. doi: 10.1016/j.addr.2016.06.007 ; PubMed Central PMCID: PMC5235994. 27327937 PMC5235994

[12] Saw PE, Song EW. Phage display screening of therapeutic peptide for cancer targeting and therapy. Protein & cell. 2019;10(11):787–807. Epub 2019/05/30. doi: 10.1007/s13238-019-0639-7 ; PubMed Central PMCID: PMC6834755. 31140150 PMC6834755

[13] Hamzeh-Mivehroud M, Mahmoudpour A, Dastmalchi S. Identification of new peptide ligands for epidermal growth factor receptor using phage display and computationally modeling their mode of binding. Chemical biology & drug design. 2012;79(3):246–59. Epub 2011/12/06. doi: 10.1111/j.1747-0285.2011.01282.x . 22136656

[14] Kim MH, Kim SG, Kim DW. A novel dual-modality imaging agent targeting folate receptor of tumor for molecular imaging and fluorescence-guided surgery. Annals of nuclear medicine. 2019;33(8):606–16. Epub 2019/05/28. doi: 10.1007/s12149-019-01369-2 . 31134434

[15] Kim MH, Kim SG, Kim DW. Dual-labeled prostate-specific membrane antigen (PSMA)-targeting agent for preoperative molecular imaging and fluorescence-guided surgery for prostate cancer. Journal of labelled compounds & radiopharmaceuticals. 2021;64(1):4–13. Epub 2020/10/11. doi: 10.1002/jlcr.3884 . 33037721

[16] Wu C, Wei J, Gao K, Wang Y. Dibenzothiazoles as novel amyloid-imaging agents. Bioorganic & medicinal chemistry. 2007;15(7):2789–96. Epub 2007/02/13. doi: 10.1016/j.bmc.2006.11.022 . 17293116

[17] Zhao X, Wang N, Ren X, Zhang J, Wang J, Han J, et al. Preparation and Evaluation of (99m)Tc-Epidermal Growth Factor Receptor (EGFR)-Peptide Nucleic Acid for Visualization of EGFR Messenger RNA Expression in Malignant Tumors. Journal of nuclear medicine: official publication, Society of Nuclear Medicine. 2014;55(6):1008–16. Epub 2014/04/20. doi: 10.2967/jnumed.113.136101 . 24744447

[18] Van de Wiele C, Revets H, Mertens N. Radioimmunoimaging. Advances and prospects. Q J Nucl Med Mol Imaging. 2004;48(4):317–25. Epub 2005/01/11. . 15640795

[19] Yang S, Shang Y, Yin S, Tian H, Chen Y, Sun S, et al. Selection and identification of single-domain antibody fragment against capsid protein of porcine circovirus type 2 (PCV2) from C. bactrianus. Veterinary immunology and immunopathology. 2014;160(1–2):12–9. Epub 2014/04/17. doi: 10.1016/j.vetimm.2014.03.004 . 24736187

[20] Sundberg AL, Gedda L, Orlova A, Bruskin A, Blomquist E, Carlsson J, et al. [177Lu]Bz-DTPA-EGF: Preclinical characterization of a potential radionuclide targeting agent against glioma. Cancer biotherapy & radiopharmaceuticals. 2004;19(2):195–204. Epub 2004/06/10. doi: 10.1089/108497804323071977 . 15186600

[21] Tolmachev V, Orlova A, Wei Q, Bruskin A, Carlsson J, Gedda L. Comparative biodistribution of potential anti-glioblastoma conjugates [111In]DTPA-hEGF and [111In]Bz-DTPA-hEGF in normal mice. Cancer biotherapy & radiopharmaceuticals. 2004;19(4):491–501. Epub 2004/09/30. doi: 10.1089/cbr.2004.19.491 . 15453964

[22] Du B, Wang S, Cui Y, Liu G, Li X, Li Y. Can (18)F-FDG PET/CT predict EGFR status in patients with non-small cell lung cancer? A systematic review and meta-analysis. BMJ open. 2021;11(6):e044313. Epub 2021/06/10. doi: 10.1136/bmjopen-2020-044313 . 34103313 PMC8190055

[23] Guo L, Chen Z, Xu C, Zhang X, Yan H, Su J, et al. Intratumoral heterogeneity of EGFR-activating mutations in advanced NSCLC patients at the single-cell level. BMC cancer. 2019;19(1):369. Epub 2019/04/25. doi: 10.1186/s12885-019-5555-y ; PubMed Central PMCID: PMC6480785. 31014278 PMC6480785

[24] Passaro A, Malapelle U, Del Re M, Attili I, Russo A, Guerini-Rocco E, et al. Understanding EGFR heterogeneity in lung cancer. ESMO open. 2020;5(5):e000919. Epub 2020/10/18. doi: 10.1136/esmoopen-2020-000919 ; PubMed Central PMCID: PMC7569934. 33067323 PMC7569934

[25] King R, Surfraz MB, Finucane C, Biagini SC, Blower PJ, Mather SJ. 99mTc-HYNIC-Gastrin Peptides: Assisted Coordination of 99mTc by Amino Acid Side Chains Results in Improved Performance Both In Vitro and In Vivo. Journal of nuclear medicine: official publication, Society of Nuclear Medicine. 2009;50(4):591–8. Epub 2009/03/18. doi: 10.2967/jnumed.108.058289 . 19289435

