# Supplementary figures and images for: A novel dual-labeled small peptide as a multimodal imaging agent for targeting wild-type EGFR in tumors
Source: PLoS One. 2022 Feb 4;17(2):e0263474. doi: 10.1371/journal.pone.0263474 (PMC8815872; doi:10.1371/journal.pone.0263474)

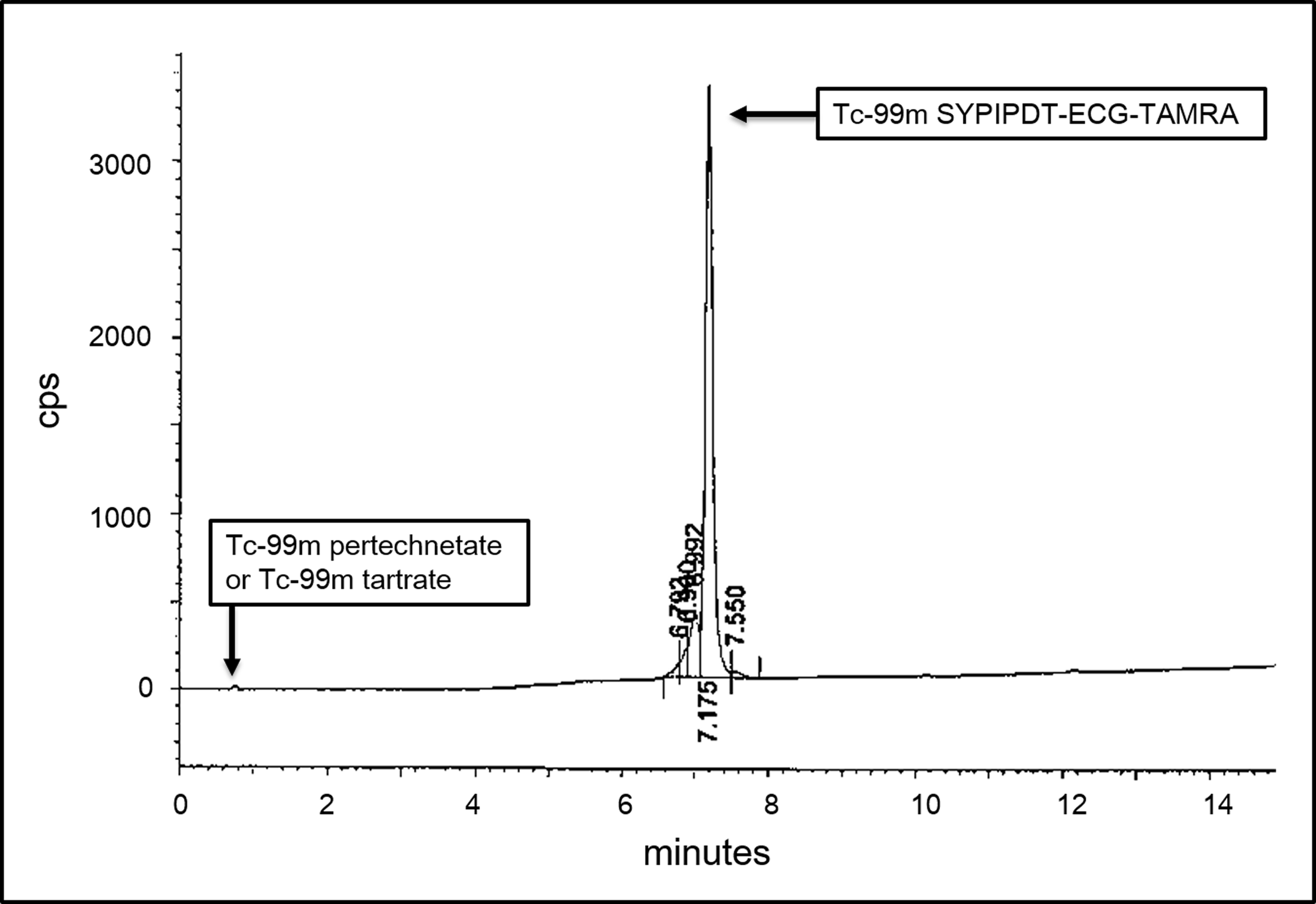

Supplement: S1 Fig — The retention time of labeled peptide was from 6.5 to 8.0 min and for mixture of Tc-99m pertechnetate and Tc-99m tartrate was 0.5–1 min. (TIF) [file pone.0263474.s001.tif]
